# Genome assembly and population genomic data of a pulmonate snail *Ellobium chinense*

**DOI:** 10.1038/s41597-023-02851-3

**Published:** 2024-01-04

**Authors:** Haena Kwak, Damin Lee, Yukyung Kim, Joohee Park, Heeseung Yeum, Donghee Kim, Yun-Wei Dong, Tomoyuki Nakano, Choongwon Jeong, Joong-Ki Park

**Affiliations:** 1 Division of EcoScience, Ewha Womans University, 52 Ewhayeodae-gil, Seodaemun-gu, Seoul, 03760 Korea; 2 School of Biological Sciences, Seoul National University, 1 Gwanak-ro, Gwanak-gu, Seoul, 08826 Korea; 3 Fisheries College, Ocean University of China, 5 Yushan Road, Qingdao, China; 4 Seto Marine Biological Laboratory, Kyoto University, 459 Shirahama, Nishimuro, Wakayama, 649-2211 Japan; 5 Natural History Museum, Ewha Womans University, 52 Ewhayeodae-gil, Seodaemun-gu, Seoul, 03760 Korea

**Keywords:** Evolutionary ecology, Ecological genetics

## Abstract

*Ellobium chinense* is an airbreathing, pulmonate gastropod species that inhabits saltmarshes in estuaries of the northwestern Pacific. Due to a rapid population decline and their unique ecological niche in estuarine ecosystems, this species has attracted special attention regarding their conservation and the genomic basis of adaptation to frequently changing environments. Here we report a draft genome assembly of *E. chinense* with a total size of 949.470 Mb and a scaffold N50 of 1.465 Mb. Comparative genomic analysis revealed that the GO terms enriched among four gastropod species are related to signal transduction involved in maintaining electrochemical gradients across the cell membrane. Population genomic analysis using the MSMC model for 14 re-sequenced individuals revealed a drastic decline in Korean and Japanese populations during the last glacial period, while the southern Chinese population retained a much larger effective population size (*N*_*e*_). These contrasting demographic changes might be attributed to multiple environmental factors during the glacial–interglacial cycles. This study provides valuable genomic resources for understanding adaptation and historical demographic responses to climate change.

## Background & Summary

Gastropods are one of the most diverse and specious molluscan classes, with some lineages having successfully radiated into diverse aquatic and terrestrial environments^1^. Recent comparative genomic analyses have provided significant insights into the adaptation of many molluscan species to different environments^2,3^, but the majority of genomic data are derived from marine or freshwater species and terrestrial/brackish water species are scarcely represented (76 marine, 24 freshwater, 1 brackish, and 5 terrestrial species in GenBank as of June 2023).

**Fig. 1:**
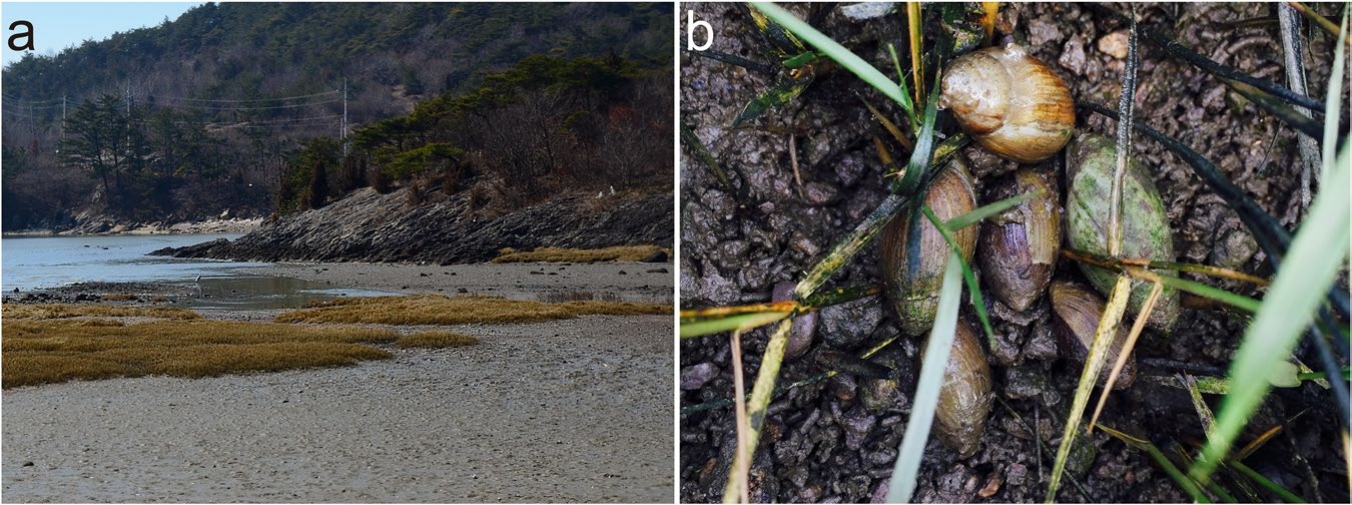
Habitat of *E. chinense*. (**a**) Habitat landscape of an estuarine saltmarsh in Korea where samples were collected. (**b**) Live individuals found in natural habitat.

**Fig. 2:**
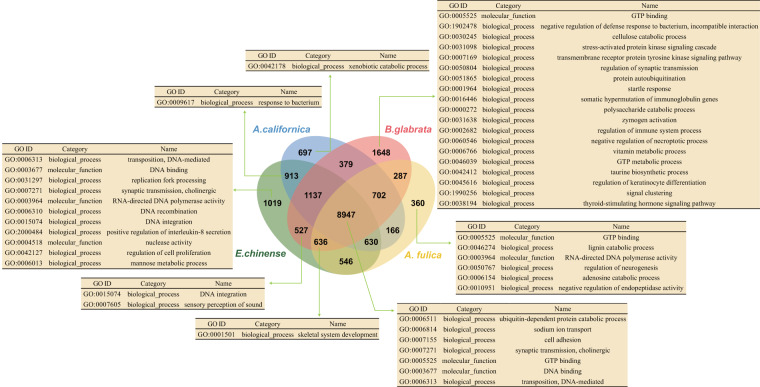
Comparative genomic analysis of orthologous genes and enriched GO terms among four gastropod species, including *E. chinense*. Venn diagram showing the unique and shared orthologous gene clusters among four gastropod species. Each table shows the list of significantly enriched GO terms (*p*-value < 0.01) identified among four gastropod species.

*Ellobium chinense* (Pfeiffer, 1854)^4^ is an airbreathing, pulmonate gastropod species that inhabits saltmarshes in estuaries of the northwestern Pacific, including Korea, Japan, and China^5^ (Fig. 1a,b). Due to a rapid population decline caused by habitat destruction from increased human activity, this species has attracted special attention regarding their conservation and is listed as Vulnerable (VU) in Korea and Japan^6,7^. Estuaries are transition zones between seas and rivers and constitute unique ecosystems, where seawater and freshwater draining from the land mix. In this respect, *E. chinense* provides an ideal model to study the genomic basis of adaptation acquired during its ecological transition (i.e., terrestrialization) from marine to nonmarine habitats^8–11^. In this study, we report the first genome sequences for this species, assembled into a draft genome of 949.470 Mb in size with a scaffold N50 of 1.465 Mb, and the results of a comparative genomic analysis of *E. chinense* with other gastropod species representing different habitat types (*Aplysia californica* [marine], *Biomphalaria glabrata* [freshwater], and *Achatina fulica* [terrestrial]). Comparative analysis of orthologous genes identified a total of 18,594 orthologous clusters, 8,947 of which were shared among four gastropod species in common and a total of 1,019 orthologous clusters were exclusively found in *E. chinense* (Fig. 2). Results from GO enrichment analysis for orthologous gene clusters revealed the top five GO terms uniquely enriched to *E. chinense* were DNA transposition (GO:0006313), DNA binding (GO:0003677), replication fork processing (GO:0031297), synaptic transmission (GO:0007271), and RNA-directed DNA polymerase activity (GO:0003964) (Fig. 2). Furthermore, the top five significantly enriched GO terms shared among four gastropod species were ubiquitin-dependent protein catabolic process (GO:0006511), sodium ion transport (GO:0006814), cell adhesion (GO:0007155), synaptic transmission (GO:0007271), and GTP binding (GO:0005525). Of these, GTP binding, synaptic transmission, and sodium ion transport are related to signal transduction that is involved in maintaining the electrochemical gradient across the cell membrane.

**Fig. 3:**
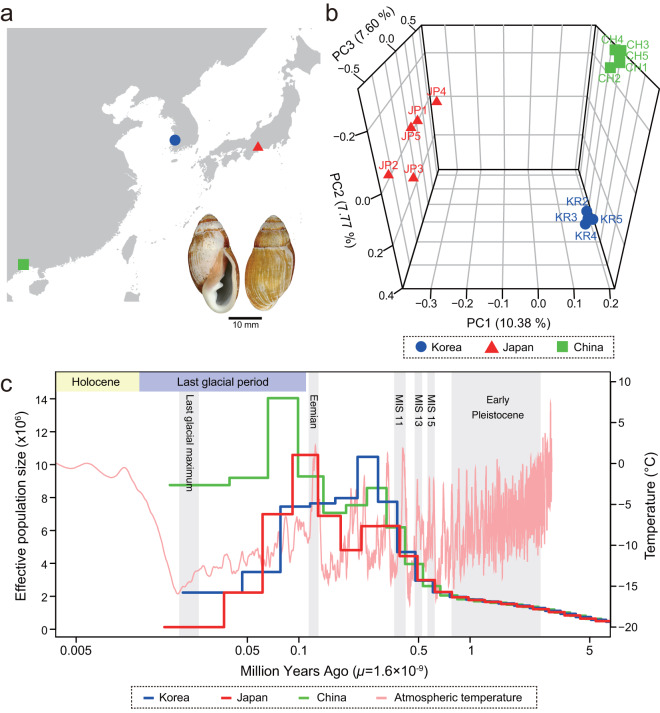
The genetic stratification and demographic history of *Ellobium chinense*. (**a**) Localities of sample collection in Japan (34°47′51.1″N, 136°33′35.2″E), China (21°37'03.1″N, 108°13′53.5″E), and Korea (35°22′51.9″N, 126°24′47.6″E). (**b**) Principal component analysis of *E. chinense* showing genetic stratification among three geographic populations. (**c**) Demographic history of the three regional populations (Korea, Japan, and China) inferred from genome sequences using MSMC2. MIS, Marine Isotope Stage.

We also performed population genomic analysis on 14 re-sequenced individuals (sequenced to ~30 X coverage) sampled from three localities (China, Japan, and Korea) (Fig. 3a) covering their native range to examine their population genetic structure and historical demographic changes. The Japanese population was genetically differentiated from the Chinese (*F*_*st*_ = 0.028) and Korean populations (*F*_*st*_ = 0.027), while there was a much lower population differentiation between Chinese and Korean populations (*F*_*st*_ = 0.005). Similarly, in our principal component analysis (PCA) based on approximately 18 Mb of genome-wide single nucleotide polymorphism (SNP) data, PC1 first separates the Japanese individuals from the Korean/Chinese individuals and PC2 successively separates the Korean individuals from the Chinese ones (Fig. 3b). We also estimated the demographic history (i.e., the trajectory of effective population size, *N*_*e*_) of *E. chinense* populations using the multiple sequentially Markovian coalescent (MSMC2 v2.11) model. Inferred *N*_*e*_ from different geographic origins showed similar demographic patterns across geographic isolates in their early stage of incremental growth until the Quaternary interglacial period of MIS 15 (Marine Isotope Stage), followed by a steep increase during the MIS 11, the longest and warmest interglacial interval, spanning between 424 kya and 374 kya (Fig. 3c). Separation of the *N*_*e*_ trajectories between populations suggests that these three regional populations split from each other after the MIS 11. Most notably, the *N*_*e*_ of the Chinese population stayed relatively high during the last glacial period, compared to the Japanese and Korean populations. The relatively high *N*_*e*_ of the Chinese population might be attributed to multiple factors, such as climatic factors, geological processes, and hydrological conditions during the glacial–interglacial cycles. The Chinese population is represented by individuals sampled from a mangrove forest in the Beibu Gulf, at the edge of the Indo-Pacific convergence region that is well known for its high biodiversity^12,13^. High temperature in this subtropical/tropical region might have played an important role in maintaining greater diversity and higher survival rates in intertidal species during glacial periods^14,15^. Since more solar radiation arrives in the tropics than at the poles, higher primary productivity may also have mediated processes that increased diversification. Furthermore, there are many subtropical–tropical islands in this region, and the extensive and diverse habitats of these peripheral islands might have provided southern Chinese populations with potential refugia during glacial periods, allowing for the maintenance of high genetic diversity^16^.

In summary, this study presents a reference genome assembly and population genomic data for *Ellobium chinense*, a pulmonated gastropod species inhabiting the saltmarshes of estuaries in the northwestern Pacific and a species of special interest for its conservation status. Comparative analysis of four gastropod draft genomes including that of *E. chinense* revealed that some commonly enriched GO terms are related to signal transduction that is involved in maintaining the electrochemical gradient across the cell membrane. A separate population genomic analysis using 14 re-sequenced individuals revealed contrasting demographic changes among studied populations (China, Japan, and Korea) during the last glacial period, that might be attributed to multiple environmental factors during the glacial–interglacial cycles. The draft genome sequence of *E. chinense* provides valuable genomic resources for understanding evolutionary adaptation, historical demographic responses to climate change, and for its future use in conservation genetics of endangered species. Nevertheless, the quality and continuity of the draft genome sequences are incomplete, thereby necessitating further investigation for its quality improvement using long-read sequencing strategy. High-quality of genome assembly from this further effort will provide a premise that can corroborate the main findings discussed in this study.

## Methods

### Sample collection and genome sequencing

For reference genome sequencing, live specimens of *E. chinense* were collected from estuarine saltmarshes in Korea (35°22'51.9“N, 126°24'47.6“E; Fig. 1a,b) under a governmental permit from the Yeongsan River Basin Environmental Office (Permit no. 2016–29). The collected samples were transferred alive to the laboratory and kept in the −80°C freezer after dissection. Total genomic DNA was extracted from foot tissue using a PCI (phenol:chloroform:isoamyl alcohol 25:24:1) solution. To construct a reference genome of *E. chinense*, we combined paired-end (180 bp, 400 bp inserts) and mate-pair (2 Kb, 5 Kb, and 8 Kb inserts) sequencing libraries on the Illumina platform (HiSeq 2000), generating a total of 118.94 Gb raw sequences accounting for approximately 125 X coverage of the final assembly (Table 1). For transcriptome sequencing, total RNA was extracted using TRIzol from the six tissues (albumen gland, digestive gland, foot, mantle, ovary, and stomach). Then, Illumina paired-end libraries with a 350 bp insert size were constructed using TruSeq RNA Sample Prep Kit v2 and sequenced on an Illumina Hiseq 4000 platform with a read length of 151 bp. Adaptor and low-quality sequences from the transcriptome data were trimmed using Trimmomatic-0.36^17^, and contaminated reads were filtered using the Kraken2 standard database^18^. The filtered transcriptome reads were then mapped to the assembled genome sequences using BWA v0.7.17^19^. The mapping rate of RNA sequence reads from six different tissue types ranged from 80.45% (stomach) to 95.41% (albumen gland) (see Supplementary Table 1 for their statistics).

**Table 1:** Sequencing and trimming statistics of genome data of *Ellobium chinense*.

Library	Raw data		Trimmomatic	Trimgalore	After error correction
	Total bps	# of reads	# of reads	# of reads	# of reads
**180 bp**	44,892,962,578	444,484,778	427,319,868	—	418,447,992
**400 bp**	44,459,634,400	440,194,400	418,636,440	—	410,601,846
**2 Kb**	11,446,638,252	111,333,052	109,813,688	98,272,594	72,795,674
**5 Kb**	9,467,237,020	93,735,020	91,447,874	82,644,038	69,735,608
**8 Kb**	8,670,825,962	85,849,762	83,247,700	76,025,676	69,466,782
**Total**	118,937,298,212	1,175,597,012	1,130,465,570	256,942,308	1,041,047,902

### Genome assembly

**Fig. 4:**
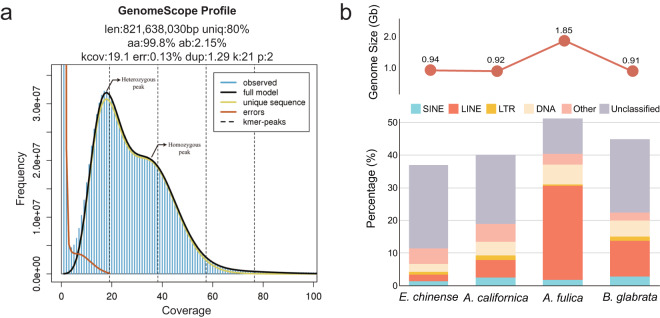
Characteristics of the *Ellobium chinense* genome. (**a**) Genome size estimation by GenomeScope2. Inferred total genome length (len); percentage of unique, non-repetitive genome (uniq); homozygosity (aa); heterozygosity (ab); mean k-mer coverage for heterozygous bases (kcov); read error rate (err); and average rate of read duplication (dup). (**b**) Comparison of genome size and repetitive sequence composition among four gastropod species, including *E. chinense*.

Raw data quality was assessed using FastQC v0.11.8^20^. Adaptor and low-quality sequences were trimmed using Trimmomatic-0.36^17^ and mate-pair libraries were trimmed again with Trimgalore v0.4.2^21^. Sequence errors in trimmed reads were corrected by a perl script, ErrorCorrectReads.pl in Allpaths-LG^22^. In all, approximately 1.04 Gb high-quality reads were generated (Table 1). A k-mer (k = 21) analysis using Jellyfish v2.3.0^23^ and GenomeScope2^24^ estimated the *E. chinense* genome size to be 822 Mb, with a heterozygosity of 2.15% which is relatively very high, compared with three other gastropod species (*A. californica* [0.962%], *B. glabrata* [1.42%], and *A. fulica* [0.138%]) (Fig. 4a and Supplementary Fig. 1). This significantly high heterozygosity level in the *E. chinense* genome sequences can lead to highly fragmented genome assembly^25^. *De novo* genome assembly of *E. chinense* was performed by Platanus (PLAT form for Assembling Nucleotide Sequences, v1.2.4)^26^. Contigs were constructed from the paired-end reads, then scaffolded and gap-closed using both paired-end and mate-pair sequences with SOAPdenovo2^27^. To avoid potential contamination from bacterial DNA, the trimmed reads with high mapping rate against bacteria sequences were removed using a BLAST search against the NCBI bacterial genome database. In the end, the *E. chinense* assembled draft genome was 949.470 Mb in size with 10,059 scaffolds and an N50 of 1.465 Mb (Table 2).

**Table 2:** Statistics of assembled genome of *E. chinense*.

	*E. chinense*
**Total length (bp)**	949,470,026
**Total length (≥50,000 bp)**	905,942,044
**Longest scaffold**	12,984,109
**# of scaffolds**	10,059
**# of scaffolds (≥50,000 bp)**	1,216
**N50 (bp)**	1,465,080
**GC content (%)**	39.38
**N content (%)**	3.32

### Repetitive sequences, gene annotation, and comparative genomic analysis

A *de novo* repeat library was generated by RepeatModeler v2.0.2^28^, and repetitive sequences were identified and masked using RepeatMasker v4.1.2^29^. Approximately 37.05% (352 Mb) of the assembled sequences of *E. chinense* were identified as repetitive sequences. Excluding the unclassified repetitive sequences (25.62%) representing the largest component in repetitive sequences, DNA transposons were the most abundant (2.42%), followed by the LINEs (2.09%), the SINEs (1.33%), and the long terminal repeat (LTR) elements (0.90%) (Table 3). Repetitive sequence composition varied greatly among the four gastropod species compared, with LINEs (long-interspersed nuclear elements) being the most conspicuously variable repetitive elements, ranging from 2.09% (*E. chinense*) to 28.92% (*A. fulica*) (Fig. 4b).

**Table 3:** Statistics of repetitive sequence of *E. chinense* genome.

	# of elements	Length occupied (bp)	Percentage (%)
**Retroelements**	185,337	40,965,477	4.31
**SINEs**	62,911	12,580,774	1.33
**LINEs**	76,150	19,864,572	2.09
**LTR elements**	46,276	8,520,131	0.90
**DNA transposons**	105,499	22,992,693	2.42
**Unclassified**	1,172,924	243,236,596	25.62
**Small RNA**	70,237	15,935,083	1.68
**Satellites**	6,895	627,397	0.07
**Simple repeats**	518,095	30,301,979	3.19
**Low complexity**	53,558	3,526,089	0.37
**Masked**		351,804,610	37.05

After excluding repetitive sequences, gene models were predicted based on a combination of homology-based and *ab initio* gene prediction approaches. For homology-based prediction, the *E. chinense* assembled genome was compared to nine metazoan species, including three non-mollusk species, from NCBI (*A. californica, B. glabrata*, *Crassostrea gigas*, *Lottia gigantea*, *Mytilus galloprovincialis*, *Octopus bimaculoides*, *Nematostella vectensis*, *Xenopus tropicalis*, and *Homo sapiens*) using the TBLASTN search. Genewise v2.4.1^30^ was used to infer gene structure based on the TBLASTN results. The transcriptome data was aligned to the assembled genome by Hisat2^31^, and *de novo* assembled by Trinity v2.4.0^32^ for *ab initio* gene model prediction. Hint files were generated by BLAT^33^ and PASA and incorporated into AUGUSTUS^34^ and GeneMark-ES^35^. EvidenceModeler combined gene prediction results and provided a consensus gene model^36^, identifying 37,866 genes in the assembled *E. chinense* genome (Table 4). Functional annotation of the predicted proteins was conducted against the NCBI NR database, the UniProtKB/Swiss-Prot database, Gene Ontology (GO), the KEGG pathway, and InterProscan. Of these identified genes, 77.40% (29,307) were assigned at least once to the databases (Table 4). For comparative genomic analysis, protein sequences from *E. chinense* and three other gastropod species inhabiting different habitats (*A. californica* [marine], *B. glabrata* [freshwater], *A. fulica* [terrestrial]) were compared. OrthoVenn2^37^, a web-based tool, was used with default parameter settings to search orthologous gene clusters and GO term enrichment, except for ortholog clustering with an e-value cutoff set to 1e-5.

**Table 4:** Statistics of functionally annotated genes of *E. chinense* genome.

		Number of genes	Percentage (%)
**Protein-coding genes**		37,866	100.00
**Annotated genes**		29,307	77.40
**Databases**	**NR**	28,730	75.87
	**UniProt**	18,914	49.95
	**InterPro**	22,035	58.19
	**GO**	15,390	40.64
	**KEGG**	8,334	22.01

### Population genomic analysis

To investigate the genetic diversity and genetic stratification of *E. chinense* populations, the whole genome was re-sequenced at ~30 X coverage for each of 14 individuals sampled from three countries covering their native range (Japan, China, and Korea). Re-sequenced reads (Supplementary Table 2) were aligned to the reference genome using BWA-mem v0.7.17^19^. The reads that mapped properly in pairs were retained using the option “−f 0 × 0003” and unmapped reads were filtered with “−F 0 × 0004” in samtools view (v1.9)^38^. PCR duplicates were removed using Picard MarkDuplicates v2.27.1, and low-quality reads (Q < 30) were filtered using samtools view. Variants were called and filtered using the Genome Analysis Toolkit (GATK) v3.8.10^39^. All sites for each individual were called by GATK HaplotypeCaller, and these per-individual gVCF files were combined into one by GATK CombineGVCFs. Then, variant sites were called by GATK GenotypeGVCFs. The biallelic SNPs with the Phred-scaled quality score ≥ 30 were kept (GATK SelectVariants), and low-quality SNPs were filtered out using GATK VariantFiltration with the following threshold; “DP < 136.0 || DP > 3400.0 || QD < 2.0 || SOR > 3.0 || FS > 60.0 || MQ < 40.0 || MQRankSum < –12.5 || ReadPosRanksum < −8.0 || ExcessHet > 10.0”. For this curated set of biallelic SNPs, another round of quality control was performed to guarantee the quality of individual genotypes. Individual genotypes were assigned as missing if the ratio of the highest genotype likelihood value to the sum of three genotype likelihoods was less than 0.99. Next, SNPs that were missing at least once in any individual were filtered out, producing a total of 36,453,320 SNPs. For most population genetic analyses, variants specific to a single individual were excluded by removing variants with (i) a minor allele count of 1 or less and (ii) doubletons with one individual homozygous for the minor allele. In the end, 18,260,324 SNPs were obtained in this variant set (18 Mb SNPs dataset). The genome coverage was estimated using QualiMap v2.21^40^. The fixation index (*F*_*st*_) was calculated by vcftools v0.1.16^41^ with the Weir & Cockerham estimator^42^. Principal component analysis (PCA) was performed on the 18 Mb SNPs dataset using smartPCA v18140 in the EIGENSOFT package v8.0.0^43^.

To estimate the demographic history of *E. chinense* populations, we used the multiple sequentially Markovian coalescent model (MSMC2 v2.1.1)^44^ based on unphased data. Input multihetsep files were generated from scaffolds larger than 1 Mb, which account for about 64% of the reference genome sequences with default parameters, using the splitfa and gen_mask programs in the SNPable package (https://lh3lh3.users.sourceforge.net/snpable.shtml) and makemappabilityMask.py, bamCaller.py, and generate_multihetsep.py scripts from the MSMC-Tools package implemented in MSMC2^44^. Then, MSMC2 was performed by pairing two haplotypes sampled from the same individual, with a default time segment parameter. To scale population parameters, we used a mutation rate estimated from *Acanthodoris* spp. (1.6 × 10^−9^ substitutions/site/generation)^45^ belonging to the Gastropoda. The generation time of *E. chinense* was set as 2 years, inferred from the life span of a closely related species, *Melampus bidentatus*^46^.

## Data Records

All DNA and RNA sequenced datasets used for genome assembly and annotation have been deposited in the NCBI Sequence Read Archive with accession numbers SRR18670280–SRR18670284^47–51^, and SRR18693111–SRR18693117^52–58^ under BioProject PRJNA824186 (DNA) and PRJNA824985 (RNA), respectively. The re-sequenced Illumina datasets used for the population genomic analyses were also deposited in the NCBI Sequence Read Archive with accession numbers SRR25445169–SRR25445182^59–72^ under BioProject PRJNA999501. The assembled genome was deposited in the NCBI with GenBank accession number JAWQUT000000000^73^. The assembled genome, predicted genes, functional annotation for comparative genomic analysis, and the BAM files and SNP data file used for population genomic analysis are available in the figshare repository, respectively^74,75^.

## Technical Validation

**Fig. 5:**
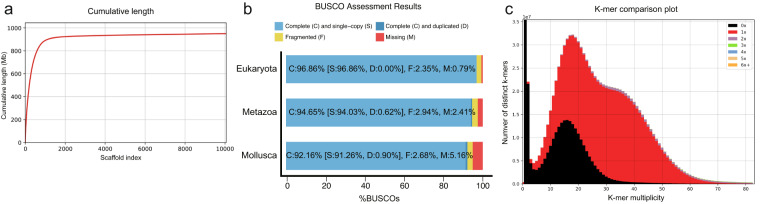
Quality assessment of assembled genome of *E. chinense*. (**a**) Cumulative length plot for aligned scaffolds by QUAST. Scaffolds ≥ 50 Kb in size account for 95.4% of the whole genome assembly. (**b**) BUSCO scores of genome assembly against three databases. (**c**) A k-mer spectra copy number plot comparing the paired-end reads to the assembled scaffolds of *E. chinense* genome.

To assess the completeness of the *E. chinense* genome assembly, filtered Illumina reads were first mapped to the assembly using BWA v0.7.17. The mapping rate of the Illumina reads was calculated with samtools flagstat (samtools v1.11) to be 97.71%. Second, QUAST v5.0.2^76^ was performed to check the assembly composition, and it was found that scaffolds longer than 50 Kb accounted for 95.4% of the total genome length (Fig. 5a). Third, genome completeness was assessed using Benchmarking Universal Single-Copy Ortholog (BUSCO) analysis with BUSCO v4.1.4^77^. The analysis was performed based on near-universal single-copy orthologs of Eukaryota, Metazoa, and Mollusca datasets (odb10) and identified 96.86% complete BUSCOs based on Eukaryota core genes, showing a high BUSCO completeness with a very low duplication rate (Fig. 5b). Finally, the assembled genome was validated by comparing it with the trimmed Illumina reads using KAT v2.4.2^78^. The KAT completeness was 54.36%, and comparison plot of k-mer spectra copy number indicated a unique haplotype genome (Fig. 5c; in red) with very low levels of duplicates (Fig. 5c; in purple). These results indicate that the genome assembly successfully collapsed diploid genome sequences to haploid genome assembly.

### Supplementary information

Supplementary Figure 1

Supplementary Table 1

Supplementary Table 2

## Data Availability

Default parameters were employed if no detailed parameters were mentioned below. (1) Trimmomatic v0.36: phred33, LEADING:3, TRAILING:3, SLIDINGWINDOW:4:15, MINLEN:36 (2) Jellyfish v2.3.0: −C −m 21 (3) GenomeScope v2: k-mer length 21, ploidy 2 (4) Population genomic analyses: All bash command lines and scripts are available at the GitHub repository: https://github.com/CWJeongLab/Ellobium, which includes detailed parameters used for population genomic analyses.
