# Supplementary material for: Genome assembly and population genomic data of a pulmonate snail Ellobium chinense
Source: Sci Data. 2024 Jan 4;11:31. doi: 10.1038/s41597-023-02851-3 (PMC10766999; doi:10.1038/s41597-023-02851-3)

**Supplementary Figure** **1.** Characteristics of the genomes of *Aplysia californica*, *Biomphalaria glabrata* and *Achatina fulica*.


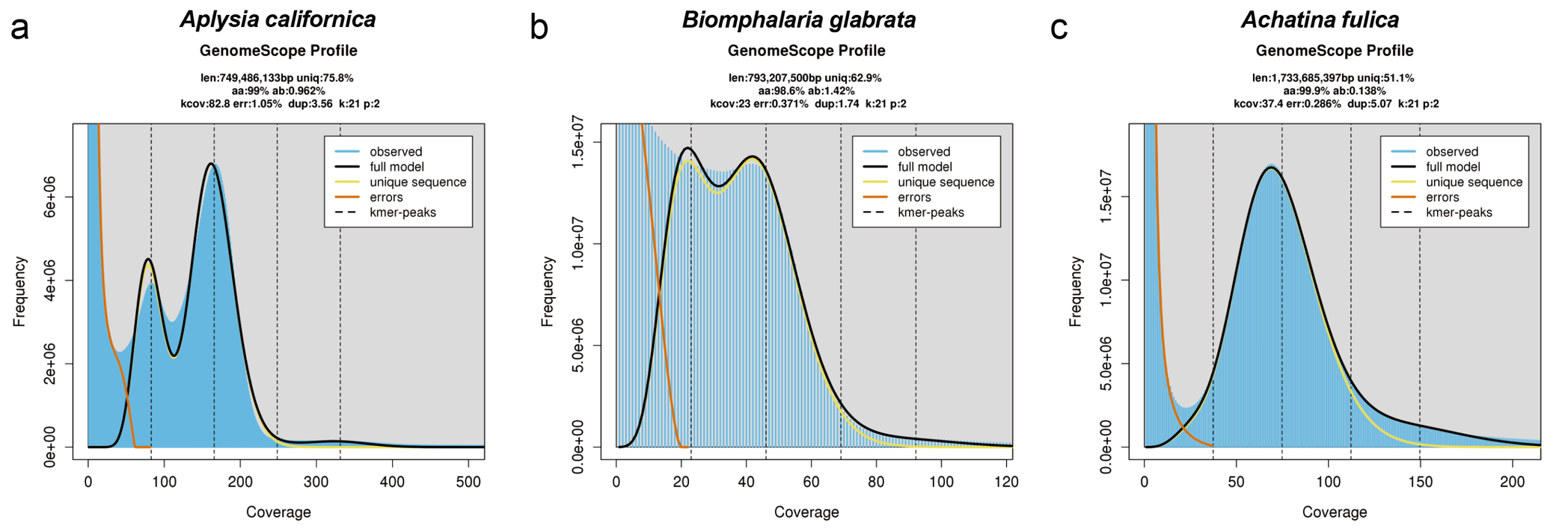

Supplement: Supplementary file 1 — Supplementary Figure 1 [file 41597_2023_2851_MOESM1_ESM.docx]
