# Supplementary material for: Genome assembly and population genomic data of a pulmonate snail Ellobium chinense
Source: Sci Data. 2024 Jan 4;11:31. doi: 10.1038/s41597-023-02851-3 (PMC10766999; doi:10.1038/s41597-023-02851-3)
Supplement: Supplementary file 2 — Supplementary Table 1 [file 41597_2023_2851_MOESM2_ESM.docx]

**Supplementary Table** **1.** Statistics of transcriptome data sequenced from six tissue types of *Ellobium chinense.*

| **Tissue** | **Raw data** | **Trimmomatic** | **Kraken2** | **BWA** |
| --- | --- | --- | --- | --- |
|  | **# of reads** | **# of reads** | **# of reads** | **Mapped %** |
| **Albumen gland** | 50,118,084 | 46,134,772 | 44,032,026 | 95.41% |
| **Digestive gland** | 52,219,968 | 48,074,896 | 46,702,724 | 85.95% |
| **Foot** | 50,855,030 | 46,585,492 | 44,440,734 | 88.32% |
| **Mantle** | 44,196,998 | 40,831,248 | 39,110,608 | 91.73% |
| **Ovary** | 47,198,402 | 43,059,712 | 41,489,466 | 91.70% |
| **Stomach** | 49,143,896 | 45,361,794 | 43,516,678 | 80.45% |
