# Supplementary material for: Genome assembly and population genomic data of a pulmonate snail Ellobium chinense
Source: Sci Data. 2024 Jan 4;11:31. doi: 10.1038/s41597-023-02851-3 (PMC10766999; doi:10.1038/s41597-023-02851-3)
Supplement: Supplementary file 3 — Supplementary Table 2 [file 41597_2023_2851_MOESM3_ESM.docx]

**Supplementary Table** **2.** Sequencing summary of re-sequenced individuals of *E. chinense*. Nr.All is total number of reads in FastQ file. Nr.Mapped is the number of reads that mapped to reference and Pr.Mapped is proportion of mapped reads out of total reads. Nr.Uniq is the number of unique reads after removing PCR duplicates. Cluster Factor is the number of mapped reads divided by the number of unique reads. Nr.q30 is the number of reads that mapping quality score is larger than 30 (q30 reads) and Pr.q30 is proportion of q30 reads out of unique reads. GC is G and C nucleotide percentage in the q30 reads. Mn.Cov and Sd.Cov are the mean and standard deviation of the coverage depth of q30 reads. Pr.Error is general alignment error rate. GC, Mn.Cov, Sd.Cov, and Pr.Error were estimated by QualiMap (v2.2.1).

| **Individual ID** | **Nr.All** | **Nr.Mapped** | **Pr.Mapped** | **Nr.Uniq** | **Cluster Factor** | **Nr.q30** | **Pr.q30** | **GC** | **Mn.Cov** | **Sd.Cov** | **Pr.Error** |
| --- | --- | --- | --- | --- | --- | --- | --- | --- | --- | --- | --- |
| Elch_CH1 | 396,585,814 | 346,346,791 | 0.873320 | 294,510,337 | 1.17601 | 271,301,269 | 0.921194 | 39.17 | 41.6578 | 30.2126 | 0.0260 |
| Elch_CH2 | 466,851,860 | 415,172,913 | 0.889305 | 332,683,719 | 1.24795 | 305,729,674 | 0.918980 | 39.19 | 46.8663 | 33.4802 | 0.0263 |
| Elch_CH3 | 531,047,682 | 460,570,670 | 0.867285 | 370,998,268 | 1.24144 | 342,471,524 | 0.923108 | 39.16 | 52.5627 | 36.2203 | 0.0261 |
| Elch_CH4 | 427,515,474 | 382,770,408 | 0.895335 | 335,545,950 | 1.14074 | 308,784,706 | 0.920246 | 39.17 | 47.3215 | 32.9583 | 0.0261 |
| Elch_CH5 | 382,978,018 | 338,911,794 | 0.884940 | 298,316,098 | 1.13608 | 275,244,863 | 0.922662 | 39.16 | 42.2599 | 30.0296 | 0.0263 |
| Elch_JP1 | 214,390,870 | 182,463,222 | 0.851075 | 153,220,444 | 1.19085 | 141,397,021 | 0.922834 | 39.17 | 21.8267 | 16.6226 | 0.0257 |
| Elch_JP2 | 405,581,016 | 354,826,745 | 0.874860 | 278,190,265 | 1.27548 | 256,455,517 | 0.921871 | 39.14 | 39.6035 | 28.5913 | 0.0257 |
| Elch_JP3 | 400,961,690 | 349,809,922 | 0.872425 | 266,520,612 | 1.31251 | 245,379,490 | 0.920677 | 39.16 | 37.8888 | 28.7592 | 0.0253 |
| Elch_JP4 | 485,128,680 | 427,405,030 | 0.881015 | 331,733,572 | 1.28840 | 305,456,361 | 0.920788 | 39.20 | 47.1545 | 34.7650 | 0.0253 |
| Elch_JP5 | 276,596,950 | 232,113,892 | 0.839175 | 194,454,540 | 1.19367 | 179,395,528 | 0.922558 | 39.17 | 27.6840 | 20.7049 | 0.0257 |
| Elch_KR1 | 440,194,400 | 399,495,738 | 0.907544 | 371,837,220 | 1.07438 | 351,546,173 | 0.945430 | 38.79 | 36.9141 | 20.6962 | 0.0111 |
| Elch_KR2 | 462,022,802 | 407,662,335 | 0.882340 | 377,457,855 | 1.08002 | 349,494,150 | 0.925916 | 39.27 | 54.0725 | 40.4165 | 0.0250 |
| Elch_KR3 | 404,613,240 | 354,379,686 | 0.875850 | 325,540,264 | 1.08859 | 301,443,064 | 0.925978 | 39.27 | 46.6108 | 33.4661 | 0.0252 |
| Elch_KR4 | 446,296,694 | 392,262,439 | 0.878930 | 361,316,811 | 1.08565 | 334,301,188 | 0.925230 | 39.29 | 51.6988 | 36.5439 | 0.0250 |
| Elch_KR5 | 551,367,870 | 485,144,989 | 0.879895 | 441,489,977 | 1.09888 | 408,496,706 | 0.925268 | 39.27 | 63.1858 | 43.8631 | 0.0253 |
